# Virilizing ovarian steroid cell tumor in a 40 year old South Indian female: a case report

**DOI:** 10.1186/1757-1626-2-7521

**Published:** 2009-05-18

**Authors:** Shihas Salim, Ghanshyam Palamaner Subash Shantha, Amish Dilip Patel, Anita A Kumar, Prasanthi Ganeshram, Nikita Mehra, Anish George Rajan, Tarun Joseph, Lavangi Sudhakar

**Affiliations:** 1 Department of General Medicine, Sri Ramachandra UniversityChennaiIndia; 2 Department of Obstetrics and Gynecology, Sri Ramachandra UniversityChennaiIndia

## Abstract

Virilism is the masculinization and enhancement of male secondary sexual characteristics in females. The etiology is usually of adrenal or ovarian origin. Here we report a case of virilizing Leydig cell type, steroid cell tumor of the left ovary, in a 40 year old female who presented with clinical signs and symptoms of virilization: deepening of voice, hirsutism (Ferriman-Gallwey score 26), clitoromegaly, and androgenic alopecia. On further evaluation, laboratory investigations revealed hyperandrogenism in the male range. Basal testosterone values were elevated. Folicle Stimulating Hormone and Luteinising Hormone levels were within normal limits. Dexamethasone suppression test did not alter cortisol or testosterone levels. An ovarian mass was confirmed radiologically. Following a total abdominal hysterectomy with bilateral salpingoophorectomy, histopathological studies confirmed a left sided steroid-cell ovarian tumor, Leydig cell type (stage T_1_N_0_M_0_), which proved to the etiology of virilization in this patient. Post-operatively her serum testosterone levels declined with near-complete reversal of symptoms over time.

## Introduction

Hirsutism is defined as excessive male-pattern hair growth. Virilization refers to a condition in which the androgen levels are sufficiently high to cause additional signs and symptoms such as deepening of the voice, breast atrophy, increased muscle bulk, clitoromegaly, and increased libido [1]. Virilizing ovarian tumors account for 0.1% of all ovarian tumors [2]. Here we report a 40-year-old woman who presented to the gynecology outpatient department with signs and symptoms of virilization. After investigations and histopathological examination, the patient was found to have a steroid-cell ovarian tumor, Leydig-cell type, which proved to the etiology of virilization in this patient.

## Case presentation

A 40-year-old multiparous woman from South India was referred to our Gynecology department with a two year history of progressive deepening of voice, an increase in facial and body hair, and frontal balding; her symptoms have worsened over the past six months. She attained menarche at the age of 14 years, and has had irregular menses lasting 3 to 4 days, every three or four months since then. She is a mother of two healthy boys, aged 12 and 15 years, both of whom were delivered vaginally without complications. The patient has been amenorrheic for the past four and a half years. There is no history suggestive of hypothyroidism or hyperprolactinemia. There is no significant family history, other than the fact that her mother attained menopause at the age of 40 years. She is a known hypertensive on treatment with calcium channel blockers. She is not on any other medications.

**Figure 1.**
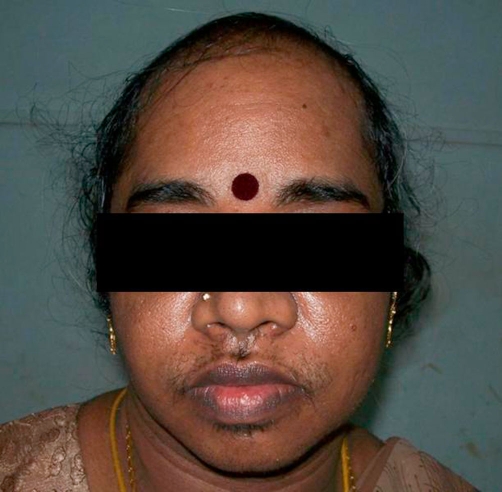
Androgenic alopecia and excess facial hair. This photo shows growth of coarse hair over the face and male pattern baldness- characteristic of virilization. Stubble is also seen over the chin and mandibular areas.

**Figure 2.**
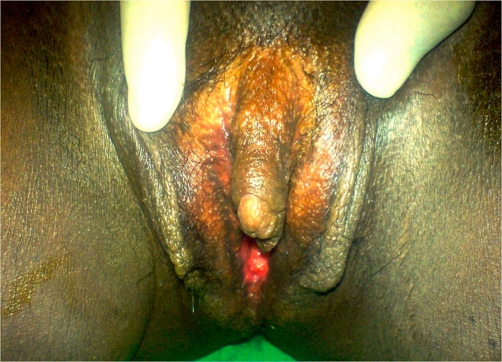
Clitoromegaly. An enlarged clitoris is visualized.

On examination, the patient was moderately built with a body mass index of 25 kg/m^2^. She had a low-pitched female voice. Head and neck examination showed male-pattern alopecia and obvious facial hair (Figure 1). There was no thyromegaly. The patient had severe hirsuties affecting the chest, forearms, thighs and anterior abdominal wall, with a Ferriman Gallwey score of 26 [3]. She was afebrile, with a pulse rate of 90 beats per minute and a blood pressure of 140/86 mmHg. Chest examination revealed bilateral breast atrophy with periareolar hair. On abdominal examination, there were no visible striae; the abdomen was soft, non-tender, with no obvious free fluid or mass on palpation. Pelvic examination revealed an enlarged clitoris (Figure 2), a nabothian follicle over the cervix, and an anteverted, small sized uterus with bilateral forniceal fullness. Other systems examination were unremarkable.

Blood investigations showed a free testosterone level of 52 ng/dl and a serum total testosterone of 2300 ng/dl (normal value: 14 - 76 ng/dl). Serum cortisol, 17-hydroxyprogesterone, prolactin, FSH, LH, thyroid hormone levels, blood sugars, and all other blood parameters were within normal limits (Table 1). Radiological imaging comprised of a chest x-ray, an ultrasonogram of the abdomen, a computed tomography (CT) scan, and a magnetic resonance imaging (MRI) scan. The chest x-ray was normal. The ultrasound of the abdomen revealed polycystic ovaries. The CT-scan of the abdomen and pelvis showed a bulky uterus (7.8 × 5.6 cm) with a mass lesion (4 × 2.1 × 3.2 cm) in the region of the fundus/left adnexa. An MRI pelvis with contrast showed two separate, well-defined oval lesions, each one adjacent to either internal iliac vessel (right: 3 × 2.5 × 2.2 cm; left: 4.3 × 3 × 2.3 cm). The lesion on the left side showed patchy enhancement after instillation of the contrast, and was seen indenting the uterine fundus. Both the ovaries were not visualized separate from either lesion. In view of the patient's age and multiparity, the management involved a staging laparotomy followed by total abdominal hysterectomy, bilateral salpingoophorectomy, and infracolic omentectomy. The specimen was sent for histopathological examination.

**Table 1.** Laboratory investigations

Parameters	Values	
Hb	12.3 g/dl	
Random blood sugar	96 mg/dl	
TSH	2.56 mIU/ml	
Estradiol	46 pg/dl	
FSH	4.3 mIU/ml	
LH	6.0 mIU/ml	
Serum Cortisol	< 1.0 mcg/dl	
17 - hydroxyprogesterone	1.2 ng/dl	
Serum Prolactin	10 ng/dl	
Serum Testosterone	Pre-operative 2300 ng/dl	Post-operative 72 ng/dl
Free Testosterone (pre-operative)	52 ng/dl	3.10 ng/dl

**Figure 3.**
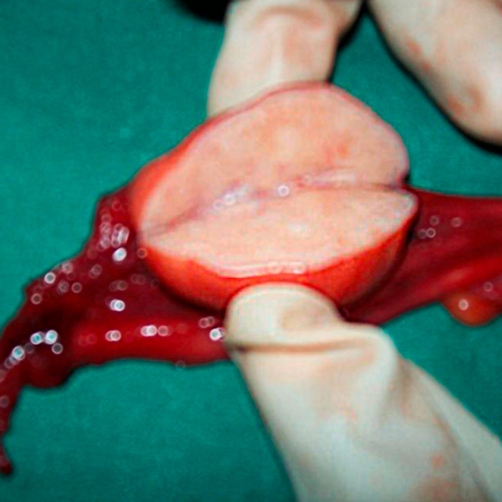
**Macroscopic appearance of the ovarian mass.** Cut section of left ovary showing a solid, grey-tan color with grey-brown patches.

**Figure 4.**
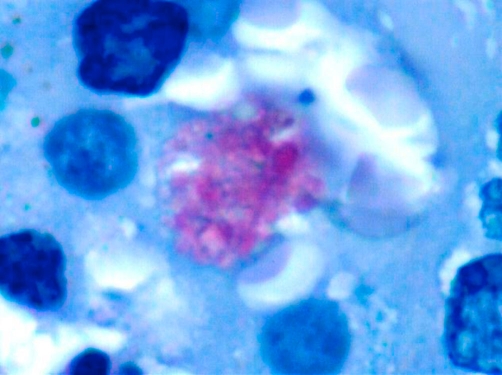
Microscopic appearance of the ovarian mass. A periodic acid-Schiff stained, histopathological specimen of the ovarian tumor (100x magnification), showing crystalloids of Reinke.

Macroscopically, both the ovaries were enlarged, tan-brown in color, and stony hard in consistency (right: 4 × 3 cm; left: 3 × 3 cm). The right ovary revealed a uniform solid grey-tan color covering the cut surface. The cut surface of the left ovary showed a solid grey-tan color with irregular grey-brown areas (Figure 3). On microscopy, there was stromal hyperplasia of both ovaries, with the left ovary showing a well differentiated steroid cell tumor. Crystalloids of Reinke were seen in plenty, thus establishing the tumor to be of Leydig cell subtype (Figure 4) [4,5]. The tumor capsule was intact and there was no evidence of vascular invasion. Peritoneal fluid cytology did not reveal any malignant cells. Intra-operatively, no significant lymph nodes were noted. Thus, tumor was staged T_1_N_0_M_0_. The preoperative elevated serum testosterone level returned to normal. There was a gradual reversal of her symptoms over time; however, the deep voice remains persistent. The patient is being followed up on a regular basis.

## Discussion

Virilizing ovarian tumors account for less than 5% of all ovarian neoplasms [6]. Sex cord-stromal tumors, are derived from the sex cord and stromal components of the developing gonad [4]. Steroid cell tumors are a subtype of ovarian sex cord-stromal tumors that are composed entirely of steroid-secreting cells and account for 0.1% of all ovarian neoplasms [2]. Steroid cell tumors are usually virilizing, frequently secreting testosterone; however, they may be endocrinologically inert or estrogenic. As many as one-quarter of steroid tumors exhibit malignant behavior [4].

Steroid cell tumors are further subclassified into stromal luteoma, Leydig cell tumors (hilar and nonhilar), and steroid cell tumors that are not otherwise specific [7]. Most pure Leydig cell tumors of the ovary arise from the hilus cells, or hilar-Leydig cells, which have the morphologic features of testicular Leydig cells and can be found in the ovarian hilus in more than 80% of adult women. These tumors are classified under the category of steroid cell tumors (lipid cell tumors) because they may be difficult or impossible to differentiate histologically from ovarian tumors of other steroid cell types [4].

The clinical manifestations are to a large extent determined by the age of presentation, hormonal activity, and virilizing properties of the tumor. Virilization or hirsutism is encountered with three fourths of Leydig cell tumors [7].

The clinical presentation may take many forms, including abdominal pain, abdominal distention, and bloating. However, the more noticeable presentations are those associated with the hormonal activity and virilizing properties of the tumor [8]. Signs and symptoms of masculinizing tumors usually take place in two definite phases, an early phase of defeminization and a subsequent phase of masculinization. Typically, a menstruating female will first notice oligomenorrhea or amenorrhea. There is regression of the breasts and external genitalia, atrophy of the uterus and adnexa, and loss of the female body contour. This is followed by hirsutism, acne, clitoral enlargement, increased libido, sterility, enlargement of the larynx, deepening of the voice, and temporal alopecia [9,10]. On the other hand, these tumors may produce little or no androgenic activity and could, in fact, show some evidence of estrogenic effect [4,10].

With regard to blood investigations, detecting the source of the androgenic tumor is a process of exclusion. In our patient, the high serum free and total testosterone confirmed the presence of a virilizing neoplasm. A dexamethasone suppression test failed to alter basal values, moreover, 17-hydroxyprogesterone levels were also within normal limits; this ruled out a potential adrenal source of the androgens [11,12].

On radiological imaging, appearances of virilizing tumors of the ovary depend, to some extent, on the tumor type. Virilizing steroid cell tumors of the ovary are usually one sided and often very small, measuring only slightly bigger than the normal ovary [9,10]. They are usually confined to the ovary at presentation, predominantly solid or mostly solid, non-calcified, and not associated with ascites. Small steroid cell tumors have been described as slightly hypoechoic or hyperechoic (compared to the ovary) with high diastolic flow on Doppler interrogation. They may be difficult to identify on radiological imaging, in part because they are isoechoic to the uterus on ultrasound and isoattenuating on CT [13,14]. Techniques such as MRI with phased array coils, or color Doppler imaging, can possibly detect smaller tumors than more conventional imaging methods [13,14,15]. In our patient, abdominal ultrasound examination was unremarkable except for the presence of ovarian cysts. A CT-scan and an MRI helped in confirming the existence of a mass lesion involving the left adnexa. It was the post-operative histopathological study, however, that helped in clinching the final diagnosis as the presence of Reinke's crystalloid is diagnostic for Leydig cell tumors [4,5,9].

Though steroid cell tumors of Leydig-cell subtype are usually benign, there is still a potential risk for malignant transformation [5,9,10]. Surgery remains the mainstay in the management of these neoplasms as, in addition to overcoming the above risk, however small, the removal of the tumor is usually followed by near-complete regression of the presenting symptoms. Initially, the signs of defeminization, such as flattened breasts and loss of fat around the hips, are reversed. Subsequent to this, the virilizing effects disappear slowly; the hypertrophied clitoris and the deepening of the voice, however, frequently persist [6,10].

## Conclusion

A patient who presents with virilism should be investigated systematically to determine if the high testosterone levels are of an adrenal or ovarian origin. In this patient, hematological investigations ruled out an adrenal cause. Radiological imaging proved to find that the cause of virilization was of an ovarian etiology. Surgical intervention was sought and the diagnosis of virilizing ovarian tumor was made by histopathologic examination.

## Patient's perspective

It was a harrowing experience for me when I started balding and growing excessive hair all over my body. My fears increased as my friends and relatives started noticing a change in my voice. When the doctors told me I had a tumor, I felt my outcome was bleak. After the operation, my physical appearance improved, and my social life has returned to normal. I am grateful to the doctors for all they have done.

## List of abbreviations

CT: Computer tomography
FSH: Follicle stimulating hormone
Hb: Hemoglobin
LH: luteinizing hormone
MRI: Magnetic resonance imaging
TSH: Thyroid stimulating hormone
